# Change in Cofactor Specificity of Oxidoreductases by Adaptive Evolution of an Escherichia coli NADPH-Auxotrophic Strain

**DOI:** 10.1128/mBio.00329-21

**Published:** 2021-08-17

**Authors:** Madeleine Bouzon, Volker Döring, Ivan Dubois, Anne Berger, Gabriele M. M. Stoffel, Liliana Calzadiaz Ramirez, Sophia N. Meyer, Marion Fouré, David Roche, Alain Perret, Tobias J. Erb, Arren Bar-Even, Steffen N. Lindner

**Affiliations:** a Génomique Métabolique, Genoscope, Institut François Jacob, CEA, CNRS, Univ Evry, Université Paris-Saclay, Evry-Courcouronnes, France; b Max Planck Institute of Molecular Plant Physiology, Potsdam-Golm, Germany; c Max Planck Institute of Terrestrial Microbiology, Marburg, Germany; d LOEWE Research Center for Synthetic Microbiology (SYNMIKRO), Marburg, Germany; Stanford University; University of Hawaii at Manoa

**Keywords:** evolution, NADPH, metabolism, NADPH-auxotrophic strain, ALE, oxidoreductases

## Abstract

The nicotinamide cofactor specificity of enzymes plays a key role in regulating metabolic processes and attaining cellular homeostasis. Multiple studies have used enzyme engineering tools or a directed evolution approach to switch the cofactor preference of specific oxidoreductases. However, whole-cell adaptation toward the emergence of novel cofactor regeneration routes has not been previously explored. To address this challenge, we used an Escherichia coli NADPH-auxotrophic strain. We continuously cultivated this strain under selective conditions. After 500 to 1,100 generations of adaptive evolution using different carbon sources, we isolated several strains capable of growing without an external NADPH source. Most isolated strains were found to harbor a mutated NAD^+^-dependent malic enzyme (MaeA). A single mutation in MaeA was found to switch cofactor specificity while lowering enzyme activity. Most mutated MaeA variants also harbored a second mutation that restored the catalytic efficiency of the enzyme. Remarkably, the best MaeA variants identified this way displayed overall superior kinetics relative to the wild-type variant with NAD^+^. In other evolved strains, the dihydrolipoamide dehydrogenase (Lpd) was mutated to accept NADP^+^, thus enabling the pyruvate dehydrogenase and 2-ketoglutarate dehydrogenase complexes to regenerate NADPH. Interestingly, no other central metabolism oxidoreductase seems to evolve toward reducing NADP^+^, which we attribute to several biochemical constraints, including unfavorable thermodynamics. This study demonstrates the potential and biochemical limits of evolving oxidoreductases within the cellular context toward changing cofactor specificity, further showing that long-term adaptive evolution can optimize enzyme activity beyond what is achievable via rational design or directed evolution using small libraries.

## INTRODUCTION

The cofactor preference of enzymes is crucial for ensuring balanced production and consumption of resources, proper regulation of metabolic processes, and general cellular homeostasis. The two primary electron carriers, NAD^+^ and NADP^+^, demonstrate this quite well, as the former is mainly involved in catabolic and respiratory processes while the latter mostly participates in biosynthetic pathways. The physiological reduction levels of NAD^+^ and NADP^+^ pools reflect this distinction. The NAD pool is highly oxidized, providing a thermodynamic push for catabolic processes, which are mostly oxidative and use NAD^+^ as an electron acceptor; in contrast, the NADP pool is relatively reduced, thermodynamically supporting anabolic processes, which are mostly reductive, using NADPH as an electron donor.

Multiple previous studies have demonstrated how the replacement of only a few residues in the active site of an oxidoreductase enzyme can switch its cofactor preference from NAD^+^ to NADP^+^ or vice versa (1–4). Several studies also constructed mutant libraries of specific oxidoreductases and harnessed natural selection to identify variants with altered cofactor specificity (5–7). These studies on changing enzyme cofactor specificities were mainly motivated by the value of enzymes as biocatalysts for industrial uses, e.g., for the production of amino acids, agrochemicals, biofuels, polymers, or pharmaceuticals. Among these oxidoreductases are enzymes active on multiple compounds, including alcohols, aldehydes, primary amines, or secondary amines. A comprehensive list of the as yet engineered oxidoreductases for NAD(P)^+^ specificity change is available in a review by Chanique et al. (2). However, until now, no study has attempted to systematically explore the overall evolvability of central metabolism oxidoreductases toward the use of a different cofactor. This could help shed light on the flexibility of the metabolic network and identify emergent regeneration processes, thus adding to our understanding of the plasticity of cellular metabolism.

Here, we applied a whole-cell evolution approach toward the emergence of new NADPH regeneration routes. We used an NADPH-auxotrophic Escherichia coli strain deleted in all enzymes capable of regenerating NADPH (Δ*zwf* Δ*maeB* Δ*icd* Δ*pntAB* Δ*sthA*), with the exception of 6-phosphogluconate dehydrogenase (8). This strain could grow on a minimal medium only when gluconate is added as an NADPH source. We conducted multiple parallel evolution experiments in continuous culture with limiting supply of gluconate, thus selecting for the emergence of mutated oxidoreductases that could reduce NADP^+^.

## RESULTS

### NADPH-auxotrophic strain and oxidoreductase candidates for NADPH regeneration.

Five enzymes are known to support NADPH regeneration in E. coli: glucose 6-phosphate dehydrogenase (Zwf), 6-phosphogluconate dehydrogenase (Gnd), the NADP^+^-dependent malic enzyme (MaeB), isocitrate dehydrogenase (Icd), and the membrane-bound transhydrogenase (PntAB) (9). In a previous study, we constructed a strain in which the genes coding for these NADP^+^-dependent oxidoreductases were deleted, with the exception of *gnd* (Δ*zwf* Δ*maeB* Δ*icd* Δ*pntAB* Δ*sthA*; the gene *sthA*, which encodes the soluble transhydrogenase, was also deleted to remove a major NADPH sink) (8). For this NADPH-auxotrophic strain to grow on a minimal medium, gluconate must be added as a precursor of 6-phosphogluconate, the substrate of Gnd. Due to the deletion of *icd*, the supply of 2-ketoglutarate as a precursor for glutamate and the downstream C_5_ amino acids glutamine, proline, and arginine is also mandatory. We demonstrated that, when gluconate is omitted, the NADPH-auxotrophic strain can serve as an effective *in vivo* platform to test and optimize different enzymatic systems for NADPH regeneration (8).

**FIG 1:**
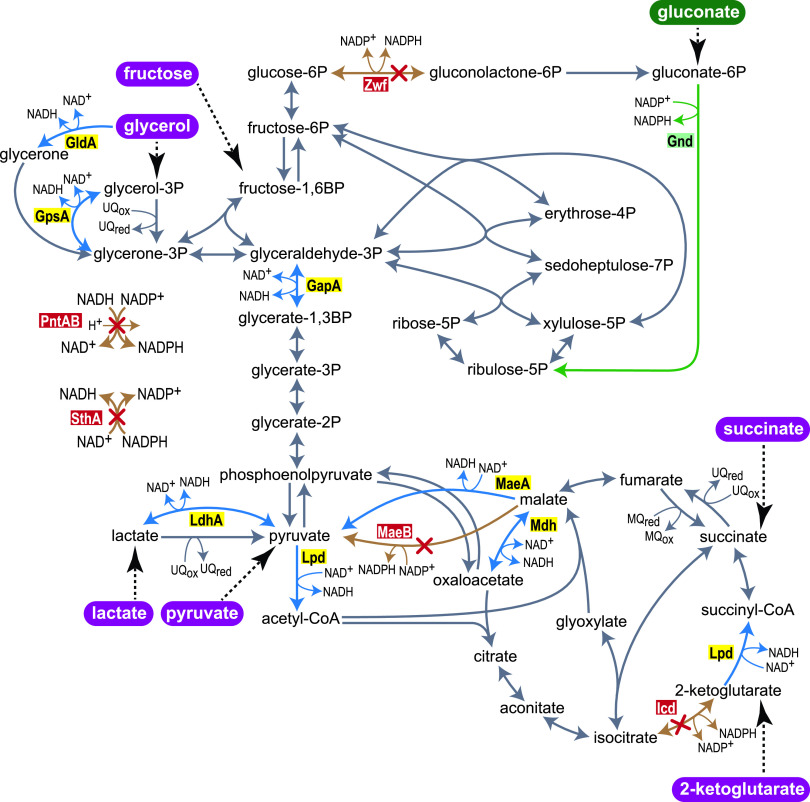
Central carbon metabolism of E. coli. NADP^+^ reducing reactions, which were deleted to construct the NADPH-auxotrophic strain, are shown by red-crossed orange arrows and red-background enzyme names. The green arrow indicates gluconate-dependent NADPH generation. Blue arrows combined with yellow-background enzyme names highlight NAD^+^-dependent oxidoreductases and potential candidates for a cofactor change from NAD^+^ to NADP^+^ in evolution experiments. Highlighted in purple are carbon sources used in evolution experiments. Zwf, glucose 6-phosphate dehydrogenase; Gnd, gluconate 6-phosphate dehydrogenase; GldA, glycerol dehydrogenase; GpsA, glycerol 3-phosphate dehydrogenase; GapA, glyceraldehyde 3-phosphate dehydrogenase; PntAB, membrane bound transhydrogenase; SthA, soluble transhydrogenase; LdhA, lactate dehydrogenase; Lpd, dihydrolipoamide dehydrogenase; MaeB, NADP^+^-dependent malic enzyme; MaeA, NAD^+^-dependent malic enzyme; Mdh, malate dehydrogenase; Icd, isocitrate dehydrogenase.

We speculated that cultivating the NADPH-auxotrophic strain under limiting amounts of gluconate would lead to the emergence of mutated oxidoreductase enzymes capable of regenerating NADPH. Such an enzyme would need to sustain very high flux to support an NADPH regeneration rate sufficiently high to enable cell growth. We therefore regarded oxidoreductase enzymes that participate in central metabolism as the major candidates for evolution toward NADP^+^ reduction. Central metabolism employs multiple oxidoreductases (Fig. 1), including glyceraldehyde 3-phosphate dehydrogenase (GapA), glycerol dehydrogenase (GldA), glycerol 3-phosphate dehydrogenase (GpsA), pyruvate/2-ketoglutarate dehydrogenases (or, more precisely, their lipoamide dehydrogenase subunit, Lpd), lactate dehydrogenase (LdhA), the NAD^+^-dependent malic enzyme (MaeA), and malate dehydrogenase (Mdh).

Since the entry point of carbon into central metabolism dictates the carbon flux distribution, we hypothesized that the choice of the carbon source could predispose different NAD^+^-dependent oxidoreductases as targets for mutations. For example, mutations in GapA that enable it to accept NADP^+^ would be useful to regenerate NADPH only if the cell is fed with a carbon source that enters upper glycolysis and induces glycolytic flux (rather than gluconeogenesis). Similarly, mutagenesis of GpsA or LdhA toward accepting NADP^+^ could effectively produce NADPH only when glycerol or lactate (respectively) serves as the carbon source.

### Adaptive evolution of the NADPH-auxotrophic strain led to mutations in *maeA* and *lpd*.

We conducted 12 evolution experiments using six carbon sources: fructose, glycerol, pyruvate, lactate, 2-ketoglutarate, and succinate (two parallel cultures for each carbon source). Fructose and glycerol are expected to force glycolytic and anaplerotic fluxes, pyruvate and lactate are expected to force gluconeogenic and anaplerotic fluxes, while 2-ketoglutarate and succinate are expected to force gluconeogenic and cataplerotic fluxes (cataplerosis being the reverse of anaplerosis, that is, decarboxylation of a C_4_ intermediate of the TCA cycle to generate a C_3_ glycolytic intermediate). Hence, the six carbon sources nicely cover a large variation in flux distribution across central metabolism (Fig. 1).

We used GM3 cultivation devices to apply a medium-swap continuous culture regime (10, 11) in order to evolve the NADPH-auxotrophic strain toward novel NADPH regeneration routes. Cultures of growing cells subjected to this regime were diluted, at fixed time intervals, by one of two growth media, permissive or stressing, the choice depending on the turbidity of the culture measured in real time. Specifically, if the turbidity was below a predefined value, a dilution pulse came from the permissive medium; otherwise, the stressing medium was used to dilute the culture (10, 11). This approach enables gradual genetic adaptation of a bacterial population to grow on the stressing medium. Here, the permissive medium contained one of the six canonical carbon sources listed above, gluconate as NADPH source, and 2-ketoglutarate as glutamate source. The stressing medium had the same composition except for gluconate, which was omitted. Continuous cultivation under these conditions was expected to select for the emergence of novel NADPH-regenerating enzymes, adapting the cells to grow with less and less gluconate, until growth on the stressing medium alone was reached.

**FIG 2:**
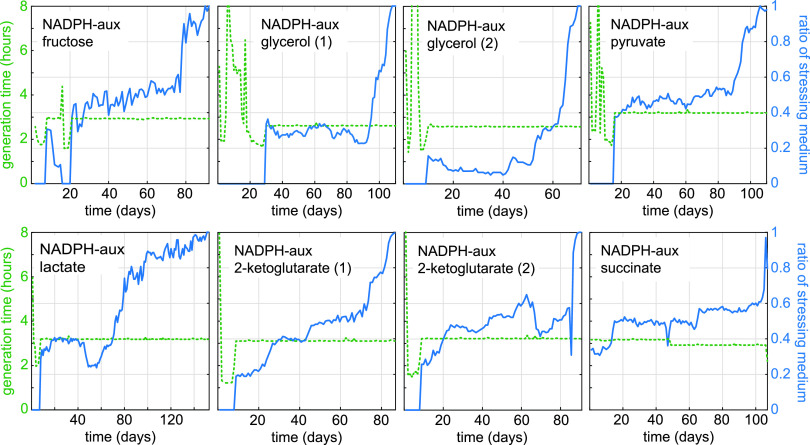
Evolution of the NADPH-auxotrophic strain for growth with either fructose, glycerol, pyruvate, lactate, 2-ketoglutarate, or succinate in the absence of gluconate. For each carbon source, two independent cultures were subjected to a medium swap regime in GM3 devices (see the Materials and Methods section). Shown are the eight cultures which evolved to grow in stressing medium. Blue lines show the ratio of stressing medium over relaxing (permissive) medium (right axis). Stressing medium contained 5 mM 2-ketoglutarate plus one of the following carbon sources: D-fructose (10 mM), succinate (17 mM), pyruvate (25 mM), lactate (25 mM), glycerol (20 mM), or 2-ketoglutarate (20 mM final). Relaxing media were composed as stressing medium supplemented with 5 mM D-gluconate. Generation times are indicated by the green dashed lines (left axes).

**FIG 3:**
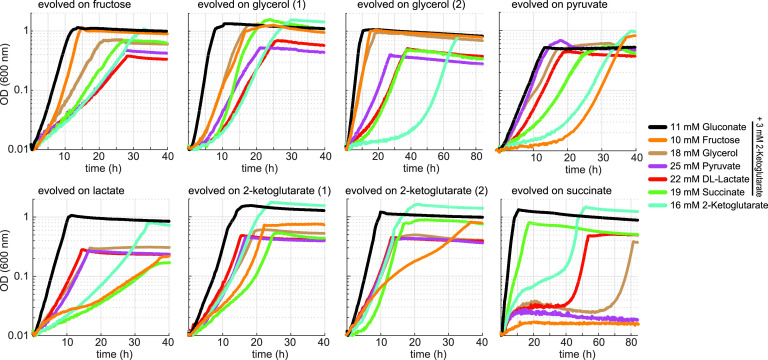
Growth curves of isolates obtained from cultures adapted in GM3 to proliferate without gluconate on either fructose, glycerol, pyruvate, lactate, 2-ketoglutarate, or succinate, each supplemented with 5 mM 2-ketoglutarate. Growth of the isolates was determined on 11 mM gluconate, 10 mM fructose, 18 mM glycerol, 25 mM pyruvate, 22 mM D/L-lactate, 19 mM succinate (all supplemented with 3 mM 2-ketoglutarate), and 16 mM 2-ketoglutarate. Growth curves were recorded in triplicates, showing similar growth (± 5%). Doubling times are available in Data Set S1.

Of the 12 parallel adaptive evolution experiments, 8 evolved to rely completely on the stressing medium (100% stressing medium pulses), including at least one culture for all six carbon sources used (Fig. 2). The adaptation kinetics and the number of generations required to attain growth without gluconate were comparable for all eight cultures (Table 1). In most cases, the stressing/relaxing dilution ratio only slightly increased during a prolonged period of the adaptation, until a sharp rise occurred and growth on the stressing medium was attained, pointing to the appearance of a key adaptive mutation in the population. We obtained single colonies from all the cultures that were evolved to grow on the stressing medium by plating a sample of the respective population on solid mineral medium having the same composition as the stressing medium. The isolated strains, besides growing on the carbon source used in the evolution experiment from which they emerged, could also grow on (almost) all other carbon sources tested, indicating that the metabolic adaptation was not restricted to a particular flux distribution in central metabolism (Fig. 3). Genome sequencing of two isolates from each successful adaptive evolution experiment was performed. Comparison with the nonevolved parent strain revealed 5 to 10 point mutations, as well as small insertions and deletions, in all genomes sequenced (Table 1). For each experiment, these isolates displayed an almost identical mutation profile (Data Set S1 in the supplemental material), suggesting that the bacterial populations in the cultures were rather homogeneous. The strains isolated from the two glycerol cultures were exceptional in that each contained more than 20 mutations, including a missense mutation in the gene *mutL*, coding for a DNA mismatch repair protein (12). Most probably, this mutation impairs efficient replication error correction, thus enabling an elevated mutation rate. However, given that the number of generations needed for these cultures to reach growth under stressing conditions was in the same range as was seen for the other cultures, we do not think the altered *mutL* gene markedly influenced the evolutionary path of the populations. In 7 of the 8 cultures, including those conducted with glycerol, the gene *maeA*, which encodes the NAD^+^-dependent malic enzyme, carried one or two nonsilent mutations. Furthermore, the isolates from the fructose and succinate cultures carried an amplified chromosomal region containing the *maeA* gene, which points to overexpression of the mutated gene as an additional adaptive trait. The only divergent isolate was from one of the cultures cultivated on 2-ketoglutarate, in which *maeA* was not mutated. Instead, *lpd*, coding for lipoamide dehydrogenase, was mutated in this strain.

**TABLE 1:** Outcome of the successful adaptive evolutions of NADPH-auxotrophic strains performed in the GM3 cultivation device; see Data Set S1 for all identified mutations

Ancestor strain	Carbon source	No. generations (until growth w/o gluconate)	No. mutations in evolved isolates	Mutated dehydrogenase	Genomic amplification
NADPH-aux (Δ*zwf* Δ*maeB* Δ*icd* Δ*pntAB* Δ*sthA*)	Fructose	700	5	MaeA D336N	+ (MaeA)
	Glycerol	750	24	MaeA D336N L176V	
		580	24	MaeA D336N L166V	
	Pyruvate	690	10	MaeA D336A I283N	
	Lactate	1,105	10	MaeA D336N S30C	
	2-ketoglutarate	609	8	Lpd E205A	
		640	10	MaeA D336N	
	Succinate	850	9	MaeA D336N	+ (MaeA)
NADPH-aux Δ*maeA* (Δ*zwf* Δ*maeB* Δ*icd* Δ*pntAB* Δ*sthA* Δ*maeA*)	Glycerol	740	9	Lpd E205A E366K^*a*^	
	Pyruvate	460	8	Lpd E205G	

a The E366K mutation was found in only one isolate from this culture. Other isolates from the same culture harbored only the E205A mutation.

10.1128/mBio.00329-21.9 DATA SET S1 Genome mutation profiles of isolated strains from evolution experiments. Doubling times of evolved and reverse engineered strains. Download Data Set S1, XLSX file, 0.02 MB. Copyright © 2021 Bouzon et al. 2021 Bouzon et al. https://creativecommons.org/licenses/by/4.0/ This content is distributed under the terms of the Creative Commons Attribution 4.0 International license.

### A mutation in a single residue in MaeA changed cofactor specificity but other mutations were essential to recover catalytic efficiency.

**FIG 4:**
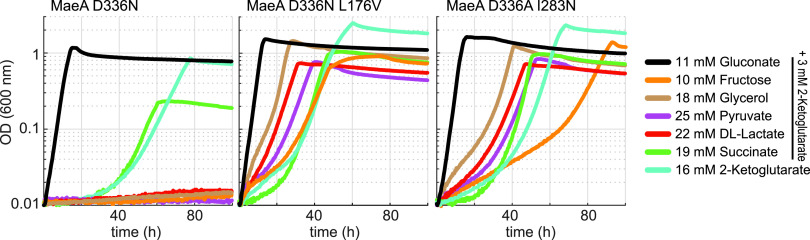
Growth of NADPH-auxotrophic strain derivatives carrying mutations in MaeA (D336N, D336N + L176V, D336A + I283N) introduced by MAGE. Growth was determined on 11 mM gluconate, 10 mM fructose, 18 mM glycerol, 25 mM pyruvate, 22 mM D/L-lactate, 19 mM succinate (all supplemented with 3 mM 2-ketoglutarate), and 16 mM 2-ketoglutarate. Growth curves were recorded in triplicates, showing similar growth (± 5%). Doubling times are available in Data Set S1.

Three of the isolated strains harbored a single mutation, D336N, in MaeA. In four strains, MaeA had two mutations, of which one was either D336N or D336A (Table 1). We chose to focus on three mutated variants: D336N, D336N L176V, and D336A I283N. We introduced each of these mutation sets into the nonevolved, parental strain using multiplex automated genomic engineering (MAGE) (13) and characterized the growth of the resulting strains (Fig. 4). As the application of the MAGE protocol might lead to additional mutations which could influence growth of the reverse engineered cells, we analyzed growth of at least three independently isolated strains. These biological replicates behaved the same in all cases.

The strain harboring MaeA D336N was able to grow only with succinate and 2-ketoglutarate but not with the other carbon sources (Fig. 4). This is in line with the fact that two of the three evolved strains displaying this mutation were cultivated on either succinate or 2-ketoglutarate, while the third one—cultivated on fructose—also showed an amplification of the chromosomal region containing the *maeA* gene (Table 1). It therefore seems that the D336N mutation enhanced NADP^+^ reduction by MaeA, but only to a limited extent. Therefore, only carbon sources that enter the TCA cycle (i.e., succinate and 2-ketoglutarate), and thus force high cataplerotic flux via MaeA, support a sufficiently high NADPH regeneration rate. When another carbon source is used (e.g., fructose) overexpression of MaeA D336N seems necessary to enable sufficient NADPH regeneration.

On the other hand, the strains harboring either MaeA D336N L176V or MaeA D336A I283N were able to grow on (almost) all carbon sources (Fig. 4). This suggested that these mutation sets increased the activity of MaeA with NADP^+^ to such a sufficiently high level that even carbon sources that do not induce cataplerotic flux could sustain a high NADPH-regeneration rate without further overexpression of *maeA*.

To test whether these interpretations were correct, we purified the mutated MaeA variants and performed steady-state analysis with NAD^+^ and NADP^+^ (Table 2). We found that while wild-type (WT) MaeA can accept NADP^+^, it uses this cofactor with a low *k_cat_*/*K_M_* = 11 s^−1 ^mM^−1^, more than 2 orders of magnitude lower than the *k_cat_*/*K_M_* of >1800 s^−1 ^mM^−1^ measured with NAD^+^. The D336N mutation lowered the *k_cat_*/*K_M_* with NAD^+^ to 49 s^−1 ^mM^−1^, while increasing *k_cat_*/*K_M_* with NADP^+^ ≈80-fold to ≈870 s^−1 ^mM^−1^. This mutation thus increased MaeA preference toward NADP—as indicated by the ratio (*k_cat_*/*K_M_*)_NADP_^+^/(*k_cat_*/*K_M_*)_NAD_^+^—by a factor of 3,000, from 0.006 to ≈18.

**TABLE 2:** Apparent steady state parameters of MaeA variants^*a*^

Enzyme	Substrate	*K_M_* (mM)	*K_cat_* (s^−1^)	*K_cat_*/*K_M_* (s^−1^ mM^−1^)	(*k_cat_*/*K_M_*)_NADP_^+^/(*k_cat_*/*K_M_*)_NAD_^+^
MaeA WT	L-malate	0.63 ± 0.04			0.0059
	NAD^+^	0.10 ± 0.02	188 ± 9	1843	
	NADP^+^	3.6 ± 0.4	39 ± 2	11	
MaeA D336N	L-malate	1.8 ± 0.2			17.6
	NAD^+^	1.5 ± 0.2	74 ± 3	49	
	NADP^+^	0.091 ± 0.01	79 ± 2	868	
MaeA D336N L176V	L-malate	1.4 ± 0.3			16.7
	NAD^+^	0.27 ± 0.03	105 ± 2	389	
	NADP^+^	0.016 ± 0.001	102 ± 1	6497	
MaeA D336A I283N	L-malate	0.48 ± 0.04			18.0
	NAD^+^	0.27 ± 0.03	130 ± 3	480	
	NADP^+^	0.013 ± 0.00	112 ± 2	8615	

a Parameters are indicated as mean value ± standard error. The underlying Michaelis-Menten kinetics can be found in Fig. S2.

10.1128/mBio.00329-21.2 FIG S2 Michaelis-Menten kinetics of MaeA variants. Data represent mean values ± SD from three independent experiments (*n* = 3). Download FIG S2, EPS file, 2.1 MB. Copyright © 2021 Bouzon et al. 2021 Bouzon et al. https://creativecommons.org/licenses/by/4.0/ This content is distributed under the terms of the Creative Commons Attribution 4.0 International license.

The combined D336N L176V and D336A I283N mutations increased the activity with NADP^+^ even more, resulting in *k_cat_*/*K_M_* values of ≈6500 s^−1 ^mM^−1^ and ≈8600 s^−1 ^mM^−1^, respectively, a 600- to 800-fold increase relative to MaeA WT. Interestingly, for these two sets of mutations, the catalytic efficiency with NAD^+^ increased to the same extent as with NADP^+^, relative to that observed in MaeA D336N. Hence, the preference of MaeA D336N L176V and MaeA D336A I283N toward NADP^+^ was effectively identical to that of MaeA D336N. It therefore seems that the main role of the L176V and I283N mutations is the recovery of the catalytic efficiency lost upon cofactor switching by the mutation of D336 (1, 14). Notably, the *k_cat_*/*K_M_* values of MaeA D336N L176V and MaeA D336A I283N with NADP^+^ are ≈4-fold higher than the *k_cat_*/*K_M_* of MaeA WT with NAD^+^, presenting one of the rare cases in which overall relative catalytic efficiency was improved upon switching the cofactor specificity.

### Upon deletion of *maeA*, adaptive evolution led to mutations in *lpd*.

**FIG 5:**
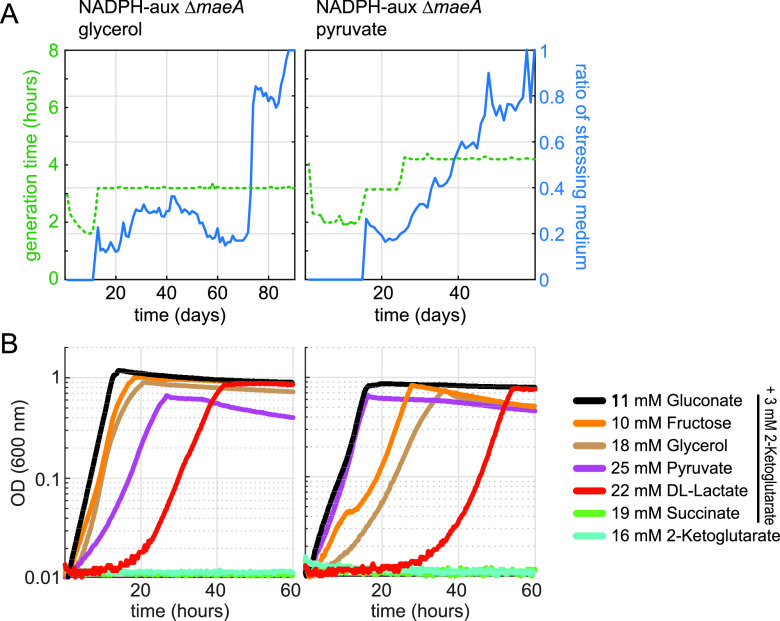
Evolution of the NADPH-auxotrophic Δ*maeA* strain for growth on glycerol or pyruvate in the absence of gluconate. For each carbon source, two independent cultures were subjected to a medium swap regime in GM3 devices (see the Materials and Methods section). For both carbon sources, one of the two cultures evolved to growth in stressing medium. (A) Evolutionary kinetics of the cultures in the GM3 device. The ratio of stressing medium (5 mM 2-ketoglutarate plus glycerol [20 mM] or pyruvate [25 mM]) over relaxing medium (same composition as stressing medium plus 5 mM D-gluconate) is shown by the blue line (right axes). Generation times are indicated by the green dashed lines (left axes). (B) Growth of isolated mutants was determined on 11 mM gluconate, 10 mM fructose, 18 mM glycerol, 25 mM pyruvate, 22 mM D/L-lactate, 19 mM succinate (all supplemented with 3 mM 2-ketoglutarate), and 16 mM 2-ketoglutarate. Growth curves were recorded in triplicates, showing similar growth (± 5%). Doubling times are available in Data Set S1.

As 7 of the 8 evolved strains contained a mutation in *maeA*, we decided to delete this gene in the NADPH-auxotrophic strain and repeat the evolution experiment in the hope to prompt the emergence of other mutations enabling NADPH regeneration. Four cultures of the Δ*zwf* Δ*maeB* Δ*icd* Δ*pntAB* Δ*sthA* Δ*maeA* strain, two supplemented with pyruvate and two with glycerol, were cultivated under the medium swap continuous culture regime described above. For both carbon sources, growth on gluconate-free stressing medium was attained for one of the two parallel cultures (Fig. 5A). The culture supplemented with pyruvate showed a rather rapid adaptation, characterized by a steady increase in the stressing/relaxing dilution pulse ratio. In contrast, the culture fed with glycerol showed a two-phase plateau-acceleration development. We isolated strains from the two cultures on the stressing medium and cultivated them on a minimal medium supplemented with different carbon sources (Fig. 5B). Both strains were able to grow on all carbon sources, with the exception of those entering the TCA cycle, i.e., on succinate and 2-ketoglutarate.

Genomic sequencing of isolates from the two cultures revealed missense mutations in *lpd* (Table 1 and Data Set S1). In all isolates, residue E205 was mutated either to glycine or to alanine; in one isolate, the E205A mutation was further accompanied by an E366K mutation. We note that the E205A mutation was also identified following the adaptive evolution of the NADPH-auxotrophic strain cultivated on 2-ketoglutarate in which MaeA did not mutate (see above, Table 1). It therefore seems that Lpd, which participates as a subunit in the pyruvate dehydrogenase and 2-ketoglutarate dehydrogenase complexes (15), was mutated to accept NADP^+^.

**FIG 6:**
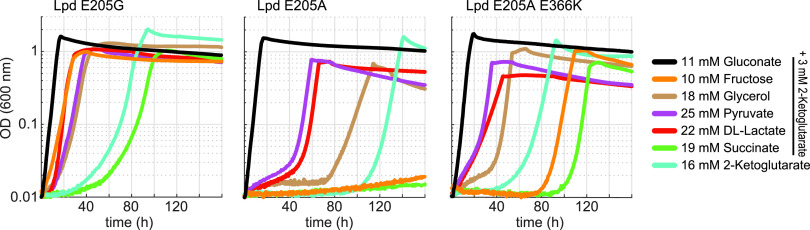
Growth of the NADPH-auxotrophic Δ*maeA* strain derivatives mutated in *lpd* (E205G, E205A, E205A + E366K) using the MAGE protocol (see the Materials and Methods section). Growth was determined on 11 mM gluconate, 10 mM fructose, 18 mM glycerol, 25 mM pyruvate, 22 mM D/L-lactate, 19 mM succinate (all supplemented with 3 mM 2-ketoglutarate), and 16 mM 2-ketoglutarate. Curves were recorded in triplicates, showing similar growth (± 5%). Doubling times are available in Data Set S1.

We used MAGE to introduce the three observed mutation sets—E205G, E205A, and E205A E366K —to the *lpd* gene in the nonevolved parental strain (NADPH-auxotrophic strain deleted in *maeA*). All resulting strains were found to grow without gluconate on (almost) all carbon sources, where the E205G mutation seemed to enable the best growth (Fig. 6). To exclude effects from potential off-target mutations caused by MAGE, we analyzed growth of at least three independently isolated strains, which behaved the same. Interestingly, while the isolated strains from the evolved culture could not grow on 2-ketoglutarate and succinate (Fig. 5B), the MAGE-constructed strains could grow on these carbon sources (Fig. 6, with the exception of Lpd E205A cultivated on succinate). Since these strains were evolved on the glycolytic carbon sources glycerol and pyruvate, the differences in growth behavior could potentially be attributed to an adaptation of the evolved strains to force anaplerotic flux rather than flux via the TCA cycle, which would be needed to grow on succinate or 2-ketoglutarate.

We further characterized the kinetics of the purified Lpd WT, Lpd E205G, Lpd E205A, and Lpd E205G E366K. We found that while Lpd WT displayed no detectable activity with NADP^+^, the mutated Lpd versions catalyzed NADP^+^ reduction with *k_cat_*/*K_M_* values of 7.8, 6.5, and 10.6 s^−1 ^mM^−1^ for Lpd E205G, Lpd E205A, and Lpd E205G E366K, respectively (Table 3). Interestingly, the preference of Lpd E205G, Lpd E205A, and Lpd E205G E366K toward NADP^+^, (*k_cat_*/*K_M_*)_NADP_^+^/(*k_cat_*/*K_M_*)_NAD_^+^ between 0.6 and 1.9, is considerably lower than that observed with the mutated variants of MaeA. And yet, such slight preference seems to ensure effective regeneration of NADPH by the mutated Lpds, thus enabling growth in a medium lacking gluconate. This might be explained by the fact that the combined flux via the pyruvate dehydrogenase and 2-ketoglutarate dehydrogenase complexes is substantially higher than that via the malic enzyme, especially since 2-ketoglutarate, as a necessary supplement (due to the *icd* deletion), was present in all experiments.

**TABLE 3:** Apparent steady state parameters of Lpd variants with NAD^+^ and NADP^+^^*a*^

Enzyme	Substrate	*K_M_* (mM)	*K_cat_* (s^−1^)	*K_cat_*/*K_M_* (s^−1^ mM^−1^)	(*k_cat_*/*K_M_*)_NADP_^+^/(*k_cat_*/*K_M_*)_NAD_^+^
Lpd WT	dihydrolipoamide	0.18 ± 0.02			
	NAD^+^	0.44 ± 0.06	363 ± 60	830	
	NADP^+^	no activity			
Lpd E205G	dihydrolipoamide	0.24 ± 0.09			0.6
	NAD^+^	9.49 ± 1.82	121.2 ± 0.9	12.8	
	NADP^+^	3.48 ± 1.03	27.2 ± 3.4	7.8	
Lpd E205A	dihydrolipoamide	0.02 ± 0.00			1.2
	NAD^+^	6.35 ± 0.38	34.3 ± 0.9	5.4	
	NADP^+^	2.48 ± 0.69	16.2 ± 2.5	6.5	
Lpd E205A E366K	dihydrolipoamide	0.03 ± 0.01			1.9
	NAD^+^	8.07 ± 0.45	45.2 ± 1.3	5.6	
	NADP^+^	3.26 ± 0.81	34.4 ± 2.9	10.6	

a Parameters are indicated as mean value ± standard error. The underlying Michaelis-Menten kinetics can be found in Fig. S3.

10.1128/mBio.00329-21.3 FIG S3 Michaelis-Menten kinetics of LPD variants. Data represent mean values ± SD from three independent experiments (*n* = 3). Download FIG S3, EPS file, 1.6 MB. Copyright © 2021 Bouzon et al. 2021 Bouzon et al. https://creativecommons.org/licenses/by/4.0/ This content is distributed under the terms of the Creative Commons Attribution 4.0 International license.

Finally, to assess the possibility that other central metabolism oxidoreductases could mutate to enable NADPH regeneration, we deleted both *maeA* and *lpd* genes in the NADPH-auxotrophic strain. We subjected the resulting strain (Δ*zwf* Δ*maeB* Δ*icd* Δ*pntAB* Δ*sthA* Δ*maeA* Δ*lpd*) to medium swap adaptation as described above, using either glycerol or succinate as a carbon source (both relaxing and stressing media were further supplemented with acetate to account for *lpd* deletion). However, even after prolonged cultivation (>1,000 generations), none of the four cultures evolved toward growth without gluconate.

## DISCUSSION

In this study, we demonstrated whole-cell adaptive evolution toward the emergence of novel routes for NADPH regeneration. In 10 successful such experiments, using different carbon sources, we found two NAD^+^-dependent oxidoreductases within central metabolism that had evolved to accept NADP^+^: the malic enzyme MaeA and lipoamide dehydrogenase Lpd.

Notably, the key residue that was mutated in all identified MaeA variants, aspartate 336 (Table 2), was also found to mutate in a previous study, enabling reductive, CO_2_-assimilating flux using NADPH as an electron donor (16). The preference toward NADP^+^ of the MaeA D336G variant described before was ≈12, somewhat lower than that observed for our mutants, i.e., 17 to 18. Moreover, the affinity of MaeA D336G toward NADP^+^, *K_M_*_,_*_app_* = 0.23 mM, was an order of magnitude lower than that observed with our mutants MaeA D336N L176V and MaeA D336A I283N, i.e., *K_M_*_,_*_app_* < 0.02 mM. This emphasizes the supportive role of the L176V and I283N mutations in restoring high catalytic efficiency upon cofactor switching. As the physiological concentration of NADP^+^ is exceedingly low, characteristically ≈1 μM in E. coli WT (17), sustaining high affinity toward this cofactor is key for its effective reduction. This explains why, in most mutated MaeA variants identified, D336 was not mutated alone, but was rather accompanied by another mutation. As mentioned above, the relative catalytic efficiency of MaeA D336N L176V and MaeA D336A I283N with NADP^+^ was found to be 4-fold higher than that of the MaeA WT with NAD^+^, thus representing one of the few cases in which cofactor switching is coupled to improved overall kinetics. An *a priori* identification of the L176 or I283 residues as targets for recovery and increase of catalytic activity would be very difficult, thus demonstrating the power of natural selection in leading to superior, yet nontrivial solutions.

The key residue that was mutated in all three Lpd variants, glutamate 205 (Table 3), was also previously mutated to switch Lpd cofactor specificity (18). In this study, the catalytic efficiency with NADP^+^ of the best engineered variant of Lpd, which harbored the E205V mutation and four additional point mutations, was 100-fold higher than that with NAD^+^ and 4-fold superior to the catalytic efficiency of the Lpd WT with NAD^+^. The consequences of the sole mutation at position 205 (E205V) on the kinetic parameters of the enzyme were not investigated. The selection of the same five combined mutations in an evolutionary experiment in continuous culture would require several events of point mutation to arise and be fixed in the same gene, which makes the occurrence of such a variant highly improbable. Comparatively, the variant Lpd enzymes selected in our experiments exhibited similar catalytic efficiencies with both nicotinamide cofactors (ratio [*k_cat_*/*K_M_*]_NADP_^+^/[*k_cat_*/*K_M_*]_NAD_^+^ between 0.6 and 1.9) and far lower catalytic efficiencies with NAD^+^ than Lpd WT. The mutations selected by the process of natural selection enlarged the cofactor specificity of Lpd with a concomitant decrease of activity, which apparently constitutes a good compromise for maintaining balanced pools of NADH and NADPH and for ensuring sufficient carbon flux to sustain growth.

Many of the evolved strains harbored a mutation in the gene coding for citrate synthase (*gltA*, Data Set S1). Interestingly, we found that even a short-term cultivation of the NADPH-auxotrophic strain using a “permissive medium,” i.e., having gluconate in the minimal medium, frequently led to mutations in this gene. These mutations included a missense mutation changing residue 150 from leucine to arginine, resulting in a 30-fold decrease in specific activity (Table S1), and a base pair deletion causing a frameshift which shortened the polypeptide from 427 amino acids (aa) to 317 aa (Data Set S1). Citrate synthase activity, while not being required for the NADPH-auxotrophic strain in the presence of 2-ketoglutarate, is probably deleterious, as it leads to overproduction and accumulation of citrate and isocitrate, which cannot be further metabolized easily due to the deletion of Icd (considering limited flux via the glyoxylate shunt). The downregulation or elimination of GltA activity avoids this overproduction and hence might be beneficial for cell growth. Comparable mutation events in citrate synthase as a consequence of TCA cycle blockage (by isocitrate dehydrogenase or aconitase deletion) were previously observed in Corynebacterium glutamicum, presumably to avoid high intracellular citrate concentrations (19).

10.1128/mBio.00329-21.6 TABLE S1 Citrate synthase activity. Download Table S1, EPS file, 1.3 MB. Copyright © 2021 Bouzon et al. 2021 Bouzon et al. https://creativecommons.org/licenses/by/4.0/ This content is distributed under the terms of the Creative Commons Attribution 4.0 International license.

It is worth noticing that out of all possible oxidoreductase enzymes in central metabolism, only MaeA and Lpd were found to mutate during the adaptive evolution. This might be explained by the fact that shifting the cofactor specificity of these two enzymes was attainable thanks to a unique point mutation. Changing the cofactor preference of the other central metabolism oxidoreductases might require the accumulation of multiple mutations, for both cofactor switching and recovery of activity (1, 14). However, a more plausible option is that the evolution of other oxidoreductases toward NADPH regeneration is biochemically constrained due to unfavorable thermodynamics. For example, the reactions catalyzed by glycerol 3-phosphate dehydrogenase (GpsA) or lactate dehydrogenase (LdhA) strongly favor NADH consumption (Δ_r_G’^m^ < −20 kJ/mol) (20). Hence, even if these enzymes were mutated to accept NADP^+^, their ability to regenerate NADPH with a sufficiently high rate—when cells are fed with the respective carbon source—would be highly limited. Indeed, glycerol and lactate catabolism involve oxidoreductases using a quinone as electron acceptor rather than NAD(P)^+^ (Fig. 1).

Similarly, if glyceraldehyde 3-phosphate dehydrogenase (GapA) would evolve to accept NADP^+^, a severe thermodynamic barrier might arise. Even with NAD^+^, substrate-level phosphorylation (involving GapA, Δ_r_G’^m^ = 24.9 kJ/mol) represents the major thermodynamic barrier in glycolysis (21). As the cellular NADP pool is substantially more reduced than the NAD pool, switching the cofactor preference of GapA from NAD^+^ to NADP^+^ would further reduce the reaction driving force, rendering glycolysis practically inoperative. Finally, the oxidation of malate to oxaloacetate is the most thermodynamically challenging reaction in central metabolism (Δ_r_G’^m^ = 30.3 kJ/mol), which can operate only if the concentration of oxaloacetate is kept very low (∼1 μM) (21). Hence, replacing NAD^+^ with NADP^+^, thus further decreasing the driving force for malate oxidation, would certainly render the TCA cycle thermodynamically infeasible. Taken together, it seems that only those central metabolism oxidoreductases which thermodynamically prefer the NAD^+^ reduction direction (MaeA, Δ_r_G’^m^ = −4.1 kJ/mol; Lpd as part of pyruvate dehydrogenase, Δ_r_G’^m^ = −35.3 kJ/mol and as part of 2-ketoglutarate dehydrogenase = −27.2 kJ/mol) could evolve to accept NADP^+^, as only these enzymes could sustain a high rate of NADPH regeneration.

Interestingly, instead of evolving a central metabolism oxidoreductase to accept NADP^+^, the adaptive evolution could have increased metabolic flux toward routes that natively produce NADPH but usually carry only low fluxes. For example, increasing flux toward serine and glycine biosynthesis and one carbon metabolism could boost NADPH regeneration via the NADP^+^-dependent bifunctional 5,10-methylene-tetrahydrofolate dehydrogenase/5,10-methenyl-tetrahydrofolate cyclohydrolase (FolD). The fact that we did not observe such adaptation indicates that it is easier to change the cofactor preference of an enzyme via few mutations rather than redistribute fluxes within the endogenous metabolic network.

Overall, the presented work shows the power of evolution and the flexibility, but also the limits of metabolism to adapt to metabolic challenges. Finding a solution counteracting the increased metabolic constraint by the additional deletion of both *maeA* and *lpd* in the NADPH-auxotrophic was not within reach in our setup, most likely because of the thermodynamic constraints of the remaining oxidoreductases, which additionally might not provide easy starting points for a cofactor switch to NADP^+^. However, the failure of the evolution attempt of the NADPH-auxotrophic Δ*maeA* Δ*lpd* indicates that this strain provides a stringent host for the *in vivo* testing of heterologous NADP^+^-specific oxidoreductases and their evolution.

## MATERIALS AND METHODS

### Reagents and chemicals.

Primers were synthesized by Eurofins (Ebersberg, Germany) (Table S2). Screening PCRs were performed using DreamTaq polymerase (Thermo Fisher Scientific, Dreieich, Germany). PCRs for amplifying deletion cassettes were done using PrimeSTAR MAX DNA polymerase (TaKaRa). NAD^+^ and NADP^+^(Na)_2_ were purchased from Carl Roth GmbH, malic acid and chicken egg lysozyme from Sigma-Aldrich AG, and DNase I from Roche Diagnostics. Dihydrolipoamide was synthesized by borohydride reduction of lipoamide (Sigma-Aldrich SA) as described by Reed et al. (22). Purity (>95%) was checked by nuclear magnetic resonance (NMR) and infusion mass-spectrometry analysis.

10.1128/mBio.00329-21.7 TABLE S2 List of DNA oligonucleotide primers used in this study; *, thioester bound. Download Table S2, EPS file, 1.9 MB. Copyright © 2021 Bouzon et al. 2021 Bouzon et al. https://creativecommons.org/licenses/by/4.0/ This content is distributed under the terms of the Creative Commons Attribution 4.0 International license.

### Media.

LB medium (1% [wt/vol] NaCl, 0.5% [wt/vol] yeast extract, 1% [wt/vol] tryptone) was used for strain maintenance. When appropriate, kanamycin (25 μg/ml), ampicillin (100 μg/ml), or chloramphenicol (30 μg/ml) was used. Minimal MA medium (31 mM Na_2_HPO_4_, 25 mM KH_2_PO_4_, 18 mM NH_4_Cl, 1 mM MgSO_4,_ 40 μM trisodic nitrilotriacetic acid, 3 μM CaCl_2_, 3 μM FeCl_3_·6H_2_O, 0.3 μM ZnCl_2_, 0.3 μM CuCl_2_·2H_2_O, 0.3 μM CoCl_2_·2H_2_O, 0.3 μM H_3_BO_3_, 1 μM MnCl_2_, 0.3 μM CrCl_3_·6H_2_O, 0.3 μM Ni_2_Cl·6H_2_O, 0.3 μM Na_2_MoO_4_·2H_2_O, and 0.3 μM Na_2_SeO_3_·5H_2_O) was used for long-term continuous cultures. M9 minimal medium (50 mM Na_2_HPO_4_, 20 mM KH_2_PO_4_, 1 mM NaCl, 20 mM NH_4_Cl, 2 mM MgSO_4_ and 100 μM CaCl_2_, 134 μM EDTA, 13 μM FeCl_3_·6H_2_O, 6.2 μM ZnCl_2_, 0.76 μM CuCl_2_·2H_2_O, 0.42 μM CoCl_2_·2H_2_O, 1.62 μM H_3_BO_3_, 0.081 μM MnCl_2_·4H_2_O) was used for cell growth analysis. The mineral media were supplemented with various carbon sources as indicated in the main text and hereafter.

### Strains and plasmids.

E. coli K12 strains used in this study are derivatives of strain SIJ488, which was used as the wild-type reference (Table 4). The deletion of the *maeA* gene was carried out by λ-Red recombination using a kanamycin resistance cassette generated via PCR using the FRT-PGK-gb2-neo-FRT (Km) cassette (Gene Bridges, Germany) and the primer pair maeA_KO_fw and maeA_KO_rv. Primers maeA_KO_Ver-F and maeA_KO_Ver-R were used to verify the deletion of *maeA* (Table S2). Cell preparation and transformation for gene deletion was carried out as previously described (23, 24). The coding sequences of the WT sequences of *maeA*, *lpd*, and *gltA*, as well as the respective mutated genes, were amplified by PCR using the primer pairs maeA_Nter_histag_fw and maeA_rv, lpd_Nter_histag_fw and lpd_rv, gltA_Nter_histag_fw and gltA-R, respectively (Table S2). The amplified fragments were inserted into a modified Novagen pET22b(+) expression vector (Table S3) by using a ligation-independent directional cloning method (25). The sequences of the inserts of the resulting plasmids were verified by Sanger sequencing.

**TABLE 4:** E. coli strains and their genetic modifications used in this study

Strain	Genotype	Reference
SIJ488	WT	24
NADPH-auxotroph	Δ*zwf* Δ*maeB* Δ*icd* Δ*pntAB* Δ*sthA*	8
NADPH-auxotroph Δ*maeA*	Δ*zwf* Δ*maeB* Δ*icd* Δ*pntAB* Δ*sthA* Δ*maeA*	This work
NADPH-auxotroph Δ*maeA* Δ*lpd*	Δ*zwf* Δ*maeB* Δ*icd* Δ*pntAB* Δ*sthA* Δ*maeA* Δ*lpd*	This work
NADPH-auxotroph *maeA*^D336N^	*maeA*^D336N^ introduced by MAGE in the NADPH-auxotrophic	This work
NADPH-auxotroph *maeA*^D336N L176V^	*maeA*^D336N L176V^ introduced by MAGE in the NADPH-auxotroph	This work
NADPH-auxotroph *maeA*^D336A I283N^	*maeA*^D336A I283N^ introduced by MAGE in the NADPH-auxotroph	This work
NADPH-auxotroph Δ*maeA lpd*^205G^	*lpd*^E205G^ introduced by MAGE in the NADPH-auxotroph Δ*maeA*	This work
NADPH-auxotroph Δ*maeA lpd*^E205A^	*lpd*^E205A^ introduced by MAGE in the NADPH-auxotroph Δ*maeA*	This work
NADPH-auxotroph Δ*maeA lpd*^E205A E366K^	*lpd*^E205A E366K^ introduced by MAGE in the NADPH-auxotroph Δ*maeA*	This work

10.1128/mBio.00329-21.8 TABLE S3 Plasmids constructed for protein purification. Download Table S3, EPS file, 1.3 MB. Copyright © 2021 Bouzon et al. 2021 Bouzon et al. https://creativecommons.org/licenses/by/4.0/ This content is distributed under the terms of the Creative Commons Attribution 4.0 International license.

### Evolution in GM3-driven long-term continuous culture.

Precultures of the NADPH-auxotrophic and NADPH-auxotrophic Δ*maeA* strains were obtained in permissive growth media consisting of minimal MA medium supplemented with 5 mM D-gluconate, 5 mM 2-ketoglutarate, and one of the following carbon sources: D-fructose (10 mM), succinate (17 mM), pyruvate (25 mM), lactate (25 mM), glycerol (20 mM), or 2-ketoglutarate (20 mM final). Each preculture was then used to inoculate the growth chambers (16 ml per chamber) of two parallel independent GM3 devices (10). A continuous gas flow of sterile air through the culture vessel ensured constant aeration and growth in suspension by counteracting cell sedimentation. The cultures were grown in the corresponding medium under turbidostat mode (dilution threshold set to 80% transmittance; optical density at 600 nm [OD_600_] ≈0.4, 37°C) until stable growth of the bacterial population. The cultures were then submitted to conditional medium swap regime. This regime enabled gradual adaptation of the bacterial populations to grow in a nonpermissive or stressing medium of composition equivalent to the permissive medium but lacking D-gluconate. Dilutions of the growing cultures were triggered every 10 min with a fixed volume of medium calculated to impose a generation time of 3 h 10 min on the cell population, if not otherwise stated. The growing cultures were fed by permissive or stressing medium depending on the turbidity of the culture with respect to a set transmittance threshold (80%, OD_600_ ≈ 0.4, 37°C). When the OD exceeded the threshold, a pulse of stressing medium was injected; otherwise a pulse of permissive medium (see Fig. S1 for a schematic representation). The cultures were maintained under medium swap regime until the bacterial cell populations grew in 100% stressing medium. Cultures which did not evolve toward growth in stressing medium were aborted after culturing for 1,000 generations. Four isolates were obtained on agar-solidified stressing medium for each successful evolution experiment and further analyzed.

10.1128/mBio.00329-21.1 FIG S1 (A) Scheme of the GM3 automated culture device operating a medium swap regime. A culture is continuously growing in a recipient and the transmittance (880 nm) measured in real time by a turbidimeter (T). A transmittance threshold is set (80%, equivalent to an OD_600_ = 0.4) and dilutions of fixed volumes are triggered at regular time intervals, either from the permissive medium (P) if T > 80% or stressing medium (S) if T < 80%. (B). Isolates from cultures evolved to grow on stressing medium are obtained by plating and their genome sequenced. For turbidostat evolution, a similar setup is used. Dilution pulses, coming exclusively from the permissive medium, are triggered each time the measured transmittance falls below the threshold. Download FIG S1, EPS file, 1.3 MB. Copyright © 2021 Bouzon et al. 2021 Bouzon et al. https://creativecommons.org/licenses/by/4.0/ This content is distributed under the terms of the Creative Commons Attribution 4.0 International license.

### Genomic analysis of evolved strains.

Pair-end libraries (2 × 150 bp) were prepared with 1 μg genomic DNA from the evolved strains and sequenced using a MiSeq sequencer (Illumina). The PALOMA pipeline, integrated in the platform MicroScope (http://www.genoscope.cns.fr/agc/microscope), was used to map the reads against the E. coli K12 wild-type strain MG1655 reference sequence (NC_000913.3) for detecting single nucleotide variations, short insertions or deletions (in/dels), as well as read coverage variations (26).

### Growth experiments.

Overnight cultures were obtained in 4 ml M9 medium supplemented with 12 mM gluconate and 3 mM 2-ketoglutarate (the permissive growth condition). Strains were harvested (6,000 × *g*, 3 min) and washed thrice in M9 medium to remove residual carbon sources. Cells were then inoculated into the various test media to OD_600_ of 0.01 and distributed into 96-well microtiter plates (Nunclon Delta Surface, Thermo Scientific). Each well contained 150 μl of culture and 50 μl mineral oil (Sigma-Aldrich) to avoid evaporation. Growth monitoring and incubation at 37°C was carried out in a microplate reader (EPOCH 2, BioTek). The program (controlled by Gen5 3.04) consisted of 4 shaking phases, 60 s each: linear shaking 567 cpm (3 mm), orbital shaking 282 cpm (3 mm), linear shaking 731 cpm (2 mm), and orbital shaking 365 cpm (2 mm). After 3 shaking cycles, absorbance OD_600_ was measured. Raw data were calibrated to 1 cm-wide cuvette measured OD_600_ values according to OD_cuvette_ = OD_plate_/0.23. Matlab was used to calculate growth parameters, repeatedly based on at least three technical replicates. Average values were used to generate the growth curves. Variability between triplicate measurements was less than 5% in all cases displayed.

### Reverse engineering.

The pORTMAGE system, which allows an efficient directed genome editing in E. coli (27), was used to introduce into the naive ancestor strains the mutations fixed in the genes *maeA* and *lpd* during the evolution experiments. MAGE oligonucleotides were designed using http://modest.biosustain.dtu.dk/ (Table S2); they contained thioester bonds at the 5′ and 3′ ends and the desired mutation. Cells carrying the pORTMAGE-2 plasmid were incubated at 30°C. When cultures reached an OD_600_ of 0.5, the system was induced by incubation at 42°C for 15 min. Afterward, cells were immediately chilled on ice until they were prepared for electroporation by 3 consecutive cycles of washing and centrifugation (11,000 rpm, 30 sec, 2°C) with ice-cold 10% glycerol (wt/vol) solution. MAGE oligonucleotides were introduced into the strains by electroporation (1 mm cuvette, 1.8 kV, 25 μF, 200 Ω). Strains were directly transferred to LB with 10 mM gluconate and 3 mM 2-ketoglutarate and incubated for 1 h. After three rounds of MAGE, cells were plated on LB plates containing 10 mM gluconate and 3 mM 2-ketoglutarate. The respective loci were amplified by PCR using respective primer pairs Ver-F and Ver-R (Table S2), and sequenced by Sanger sequencing to identify strains with the desired mutations.

### Protein expression and purification.

The His-tagged WT and MaeA variants were expressed in E. coli strain BL21(DE3). Cells were grown in Terrific Broth containing 100 μg/ml ampicillin at 37°C until they reached an OD_600_ of 0.8 to 1, upon which expression for 16 h at 23°C was induced by addition of 250 μM IPTG (isopropyl-D-β-thiogalactopyranoside). Cells were harvested by centrifugation for 15 min at 6,000 × *g* at 4°C and then resuspended in 2 ml of Buffer A (50 mM Tris, 500 mM NaCl, pH 7.5) per gram of pellet. The suspension was treated with 10 mg/ml of DNase I, 5 mM MgCl_2_, and 6 μg/ml lysozyme on ice for 20 min, after which cells were lysed by sonication. The lysate was clarified at 45,000 × *g* at 4°C for 45 min and the supernatant was filtered through a 0.4-μm syringe tip filter (Sarstedt, Nümbrecht, Germany). Lysate was loaded onto a pre-equilibrated 1 ml HisTrap FF column and washed with 12% Buffer B (50 mM Tris, 500 mM NaCl, 500 mM imidazole, pH 7.5) for 20 to 30 column volumes until the UV 280 nm reached the baseline level. The protein was eluted by applying 100% buffer B, collected, then pooled and desalted into 12.5 mM Tris, 125 mM NaCl, pH 7.5, 10% (wt/vol) glycerol. Protein purity was assessed by SDS-PAGE (Fig. S4) and its concentration was determined on a NanoDrop at 280 nm. The protein was frozen in liquid nitrogen and stored at −80°C if not immediately used for assays.

10.1128/mBio.00329-21.4 FIG S4 SDS page of purified MaeA variants. Download FIG S4, EPS file, 2.7 MB. Copyright © 2021 Bouzon et al. 2021 Bouzon et al. https://creativecommons.org/licenses/by/4.0/ This content is distributed under the terms of the Creative Commons Attribution 4.0 International license.

The His-tagged WT and mutated Lpd proteins were expressed in E. coli BL21(DE3) Codon+ (Invitrogen). Cells in 400 ml Terrific Broth containing 0.5 M sorbitol, 5 mM betaine, and 100 μg/ml carbenicillin were grown at 37°C until they reached an OD_600_ of 0.8 to 1, upon which expression for 16 h at 20°C was induced by addition of 500 μM IPTG. Cells were harvested by centrifugation for 30 min at 10,000 × *g* at 4°C. Cell pellets were frozen at −80°C for one night. Thawed cells were then suspended in 32 ml of Buffer A (50 mM phosphate [Na/K], 500 mM NaCl, 30 mM imidazole, 15% [wt/vol] glycerol, pH 8.0) and lysed for 30 min at room temperature after addition of 3.6 ml of Bug Buster (Novagen), 32 μl of 1 M DTT (dithiothreitol), 320 μl Pefabloc 0.1 M (Millipore), and 23 μl Lysonase (Novagen). Lysate was clarified at 9,000 × *g* for 45 min at 4°C and then loaded onto a 5-ml HisTrap FF column (GE Life Sciences) pre-equilibrated in Buffer A. The protein was eluted in Buffer B (50 mM phosphate [Na/K], 500 mM NaCl, 250 mM imidazole, 1 mM DTT 15% [wt/vol] glycerol, pH 8.0) and further purified on a gel-filtration column Hi Load 16/60 Superdex 200 pg in Buffer C (50 mM Tris, 50 mM NaCl, glycerol 15% [wt/vol], 1 mM DTT, pH 8.0). Expected size and purity of the proteins were checked by sodium dodecyl sulfate-polyacrylamide gel electrophoresis with NuPage system (Invitrogen) and molecular weight standards (SeeBlue Plus2, Invitrogen) (Fig. S5). The protein was frozen and stored at −80°C if not immediately used for assays.

10.1128/mBio.00329-21.5 FIG S5 SDS page of purified Lpd variants. Download FIG S5, EPS file, 1.8 MB. Copyright © 2021 Bouzon et al. 2021 Bouzon et al. https://creativecommons.org/licenses/by/4.0/ This content is distributed under the terms of the Creative Commons Attribution 4.0 International license.

### Biochemical assays.

**Characterization of MaeA kinetic parameters.** Assays were performed on a Cary-60 UV/Vis spectrophotometer (Agilent) at 30°C using quartz cuvettes (10 mm path length; Hellma). Reactions were performed in 50 mM Tris HCl (pH 7.5) and 10 mM MgCl_2_. Kinetic parameters for one substrate were determined by varying its concentration (l-malate 500 μM to 20 mM, NAD^+^ 62.5 μM to 8 mM, NADP^+^ 15.2 μM to 10 mM) while the others were kept constant at 6 to 10 times their *K_M_* value. The reaction was started by addition of enzyme at final concentrations between 15 nM and 136 nM. Reaction procedure was monitored by following the reduction of NAD(P)^+^ at 340 nm (ε_NADPH,340nm_ = 6.22 mM^−1 ^cm^−1^). Each concentration was measured in triplicates and the obtained curves were fit using GraphPad Prism 8. Hyperbolic curves were fit to the Michaelis-Menten equation to obtain apparent *k_cat_* and *K_M_* values.

**Characterization of Lpd kinetic parameters.** Assays were performed using a Safas UV mc2 double beam spectrophotometer at room temperature using quartz cuvettes (10 mm path length). The concentration of purified Lpd enzyme was determined spectrophotometrically using an extinction coefficient of 34.0 mM^−1^ cm^−1^ at 280 nm (computed using Expasy Paramtool https://web.expasy.org/protparam/). The concentration of FAD was determined using an extinction coefficient of 15.4 mM^−1^ cm^−1^ at 446 nm (28). The absorbances (A) at 446 nm and at 280 nm were measured and the ratio A_446nm_/A_280nm_ calculated to determine the fraction of active FAD-containing catalysts within each batch of purified enzyme and to normalize the results between the different enzyme forms. FAD-containing Lpd ratios varied from 8% to 10% of total protein. Assays of Lpd-catalyzed oxidation of dihydrolipoamide were conducted in 100 mM Na phosphate, 100 mM KCl, and 8 mM Tris(2-carboxyethyl)phosphine hydrochloride (TCEP) pH 7.6. Kinetic parameters for NAD(P)+ were determined by varying its concentration (0, 0.5, 1, 2, 4, 6, 10, and 20 mM) in the presence of a saturating concentration of dihydrolipoamide (4 mM) and final enzyme concentrations varying from 0.03 to 3 μM. The reactions were monitored by recording the linear accumulation of NAD(P)H at 340 nm. Kinetic constants were determined by nonlinear analysis of initial rates from duplicate experiments using SigmaPlot 9.0 (Systat Software, Inc.).

### Data availability.

The data supporting the findings of this work are available within the paper and its supplemental material files. Strains used here are available on request from the corresponding author. For ΔG calculations, we used data from eQuilibrator (http://equilibrator.weizmann.ac.il/).

10.1128/mBio.00329-21.10 TEXT S1 Supplementary methods. Download Text S1, DOCX file, 0.02 MB. Copyright © 2021 Bouzon et al. 2021 Bouzon et al. https://creativecommons.org/licenses/by/4.0/ This content is distributed under the terms of the Creative Commons Attribution 4.0 International license.
