# Supplementary material for: Change in Cofactor Specificity of Oxidoreductases by Adaptive Evolution of an Escherichia coli NADPH-Auxotrophic Strain
Source: mBio. 2021 Aug 17;12(4):e00329-21. doi: 10.1128/mBio.00329-21 (PMC8406311; doi:10.1128/mBio.00329-21)
Supplement: TEXT S1 [file mbio.00329-21-s0001.docx]

**Supplementary Methods**

**GltA expression and purification.** The His-tagged WT and L150R-mutated GltA proteins were expressed in *E. coli* BL21 DE3 Codon+ (Invitrogen). Cells in Terrific broth containing 100 µg/mL carbenicillin were grown at 37 °C until they reached an OD_600_ = of 0.8-1 upon which expression for 16 h at 20 °C was induced by addition of 500 μM IPTG. Cells were harvested by centrifugation for 30 min at 10000g at 4°C. Cell pellets were frozen at -80°C for one night. Thawed cells were then suspended in 3 ml of Buffer A (50 mM HEPES, 50 mM NaCl, 30 mM imidazole, 10 % (w/v) glycerol, pH 8.0) and incubated with Pefabloc and Lysonase for 20 min then lysed by sonication. The lysate was clarified at 12000 g for 30 min at 4 °C. The supernatant was loaded onto a pre-equilibrated Ni-NTA minicolumn (QIAGEN) and washed thrice with Buffer A. The protein was eluted in elution buffer B (50 mM HEPES, 50 mM NaCl 250 mM imidazole, 10 % (w/v) glycerol), collected, pooled and desalted on an Amicon Ultra-4 10kD column in buffer C (50 mM HEPES 50 mM NaCl 10 % (w/v) glycerol). The protein was frozen and stored at -80°C if not immediately used for assays.

**Measurement of GltA specific activity.** Citrate synthase activity was determined by measuring the initial rate of reaction at 412 nm by means of the DTNB method [1]. Reactions were conducted in 100 mM Tris pH 8, 200 µM DTNB, sub-saturating concentrations of acetyl-CoA (200 µM) and oxaloacetate (100 µM) and with or without the addition of NADH (200 µM) and KCl (100 mM). Specific activity (µmole min^-1^ mg^-1^) was determined.

**Supplementary reference**

1. Moriyama, T. & Srere, P. A. (1971) Purification of rat heart and rat liver citrate synthases. Physical, kinetic, and immunological studies, *J Biol Chem.* **246**, 3217-23.
